# One genome, multiple phenotypes: decoding the evolution and mechanisms of environmentally induced developmental plasticity in insects

**DOI:** 10.1042/BST20210995

**Published:** 2023-03-16

**Authors:** Kane J. Yoon, Christopher B. Cunningham, Amanda Bretman, Elizabeth J. Duncan

**Affiliations:** 1 School of Biology, Faculty of Biological Sciences, University of Leeds, LS2 9JT Leeds, U.K.; 2 Department of Entomology, University of Georgia, Athens, GA 30602, U.S.A

**Keywords:** developmental plasticity, evo-devo, insect, neuroendocrine, polyphenism

## Abstract

Plasticity in developmental processes gives rise to remarkable environmentally induced phenotypes. Some of the most striking and well-studied examples of developmental plasticity are seen in insects. For example, beetle horn size responds to nutritional state, butterfly eyespots are enlarged in response to temperature and humidity, and environmental cues also give rise to the queen and worker castes of eusocial insects. These phenotypes arise from essentially identical genomes in response to an environmental cue during development. Developmental plasticity is taxonomically widespread, affects individual fitness, and may act as a rapid-response mechanism allowing individuals to adapt to changing environments. Despite the importance and prevalence of developmental plasticity, there remains scant mechanistic understanding of how it works or evolves. In this review, we use key examples to discuss what is known about developmental plasticity in insects and identify fundamental gaps in the current knowledge. We highlight the importance of working towards a fully integrated understanding of developmental plasticity in a diverse range of species. Furthermore, we advocate for the use of comparative studies in an evo-devo framework to address how developmental plasticity works and how it evolves.

## Introduction

‘Phenotypic plasticity’ is the ability of identical genotypes to produce different phenotypes in response to environmental cues [1–3]. If this environmental cue affects or influences an organism's development, it is called ‘developmental plasticity’. Development, the period between gamete fusion and final adult form, is when highly stable phenotypic trajectories are established, having implications for the rest of the organism's life. Developmental plasticity is widespread among plants and animals and occurs in response to various environmental cues [4–15].

Developmental plasticity can be adaptive, improving survival and reproduction [16]. For example, the seasonal variation in wing eyespot pattern in *Bicyclus anynana* [6] increases predator avoidance [17] and has a role in mate selection [18]. However, developmental plasticity is largely irreversible which can lead to mismatches between the phenotype and the environment if the environment is rapidly changing. This mismatch may then have maladaptive consequences. An example of this is seen in the crustacean *Daphnia* spp. where the development of anti-predator defensive structures comes at the cost of reduced fecundity [19]. If *Daphnia* with the defensive phenotypes hatch into an environment without predators, they will be at a selective disadvantage because of this mismatch between their developmental trajectory and actual environmental conditions. Mismatch has been linked in humans to risks of developing metabolic diseases, including obesity, type II diabetes and cardiovascular disease [20,21].

The suggestion that plasticity may have a significant role for evolution was first made by West-Eberhard [3,22]. However, the evidence for whether plasticity hinders or facilitates adaptive evolution is somewhat contradictory and may depend on how fast the environment changes and how variable the environment is [23]. Developmental plasticity is, therefore, relevant for a range of research fields. In conservation biology, a central question is whether developmental plasticity aids or retards species’ adaptation to the rapidly changing global climate [23]. In sustainable food production, it is relevant to ask whether plasticity could be manipulated to increase crop yield [24]. In medicine, it is important to determine if developmental plasticity can be ‘reprogrammed’ to alleviate the public health consequences of later-life metabolic disease [20,21]. To address these questions, it is imperative to understand how developmental plasticity works; what genetic processes underlie how organisms respond to environmental cues, how does the environmental cue modify developmental mechanisms, and how are the alternative phenotypes encoded in the genome? Importantly, exploring these questions will allow us to address the evolutionary mechanisms and consequences of developmental plasticity.

**Figure 1.**
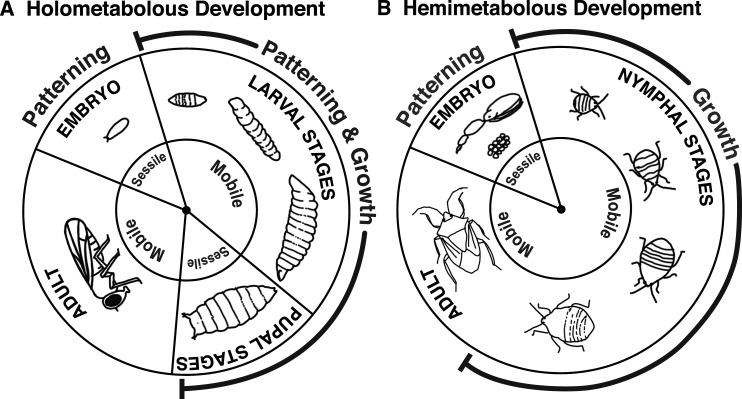
Key stages of insect life cycles. Stages of insect life cycles in (**A**) holometabolous (modified from Mirth et al. [16]) and (**B**) hemimetabolous insects. Holometabolous insects go through a complete metamorphosis. The adult structures form from imaginal discs, which grow and are patterned during larval development. In hemimetabolous insects, there is an incomplete metamorphosis. Patterning and growth are largely uncoupled, with patterning and some growth occurring during embryonic development and most growth occurring during post-embryonic development. These life cycle stages are differentially sensitive to environmental conditions and developmental plasticity.

**Figure 2.**
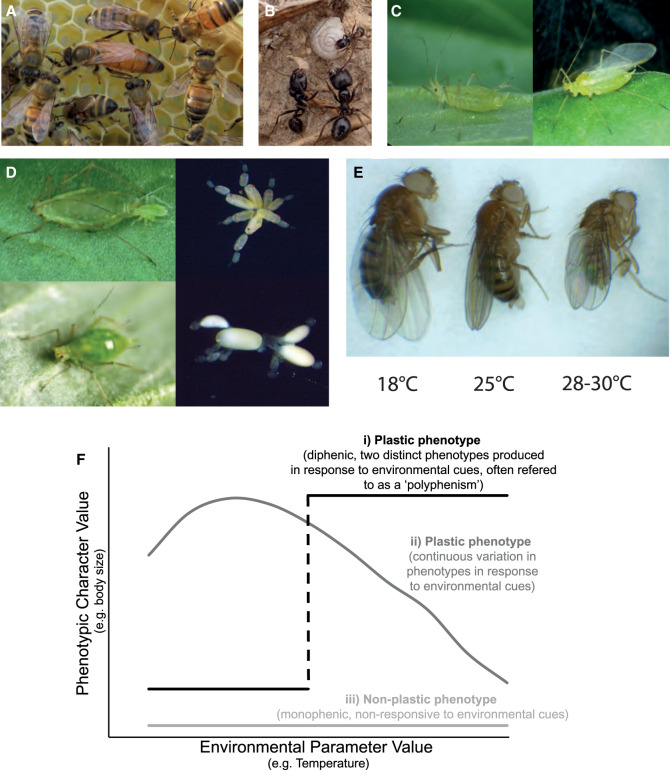
Well-known examples of developmental plasticity in insects (Images A–F Authors’ own work). (**A**) Queen (centre) and worker castes of the honeybee (Apis mellifera) are both female and can develop from genetically identical larvae in response to differential nutrition. (**B**) Plasticity is also seen amongst worker castes of some eusocial insects (pictured are soldier [bottom] and worker [top] castes of the harvester ant Messor barbarus). (**C**) Wing development is responsive to crowded conditions in aphids (pictured is the pea aphid, Acyrthosiphon pisum). (**D**) Aphids, including the pea aphid, exhibit plasticity in ovary development. Long-day length and higher temperatures yield aphids that reproduce asexually that give birth to live young. When day length shortens and temperatures decrease, ovary development is altered, and females give birth to aphids that reproduce sexually and lay eggs. (**E**) Body size in most insects (including Drosophila melanogaster, pictured) is responsive to developmental temperature. (**F**) Stylised graph demonstrating the relationship between phenotype and environmental cues for different kinds of developmental plasticity. (i) Illustrates polyphenisms, where only two discrete phenotypes are generated in response to the environmental cue. Polyphenisms are common in insects, and A–D are examples of polyphenisms. (ii) Continuous plasticity, where the phenotype varies continuously with the environmental cue (as illustrated in **E**). (iii) A non-plastic trait that is not responsive to an environmental cue. The graph was adapted from [123], and the data for the continuous plastic trait (ii) was obtained from [124] using WebPlotDigitizer (https://automeris.io/WebPlotDigitizer/index.html).

In this review, we focus on developmental plasticity in insects. Insects are integral to ecosystem function, and insect numbers are in steep decline globally [25–27]. Despite this general pattern, a small number of species are thriving [25], and it has been proposed that developmental plasticity may facilitate the success of these species [28]. Insects are the most speciose animal class, with an estimated 5.5 million species [29]. Integral to their evolutionary success was the evolution of metamorphosis, giving rise to holometabolous insects (Figure 1), which includes flies, beetles, moths, butterflies, bees, ants, and wasps. Both holometabolous (complete metamorphosis) and hemimetabolous (incomplete metamorphosis) insects are highly sensitive to environmental cues throughout development (Figure 1). Insects exhibit some of the most well-studied and tractable examples of developmental plasticity (Figure 2). Notable examples of developmental plasticity include the queen-worker and worker-worker caste polyphenisms of social insects (Figure 2A,B), solitary-gregarious forms of locusts, seasonal wing colouration patterns of *B. anynana*, and the wing and reproductive polyphenisms of aphids (Figure 2C,D). Developmental plasticity is also seen in a range of other insect traits, including body size in response to developmental temperature (Figure 2E). Polyphenisms are special cases of developmental plasticity where an environmental cue causes two or more discrete classes of phenotypes (e.g. queen and worker castes) rather than continuous variation in a trait (e.g. body size; Figure 2F). Much of the research to date on the mechanisms and evolution of developmental plasticity has focussed on polyphenisms.

## How does developmental plasticity work?

**Figure 3.**
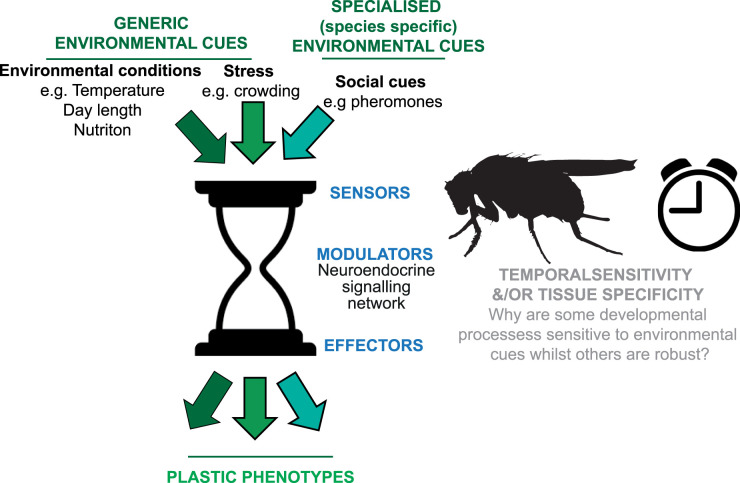
Mechanisms of developmental plasticity. Alternative phenotypes are induced in insects in response to a range of environmental stimuli. For developmental plasticity to function, insects must be able to (a) detect these stimuli using specialised sensors, (b) transmit this information to the affected tissue via a very limited range of modulators (relative to the diversity of cues, sensors and effectors) such as hormones and (c) the affected tissue must alter gene expression in response to these modulators, to induce an alternative developmental trajectory and a plastic phenotype (effector modules). This diagram highlights two key areas where we lack understanding at present: firstly, how are a diverse range of environmental cues relayed through a very limited set of modulators (primarily the hormones insulin, juvenile hormone and ecdysone) to give rise to a range of different plastic phenotypes and secondly, how temporal or tissue-specific developmental plasticity is modulated. Together, answering these questions will allow us to determine the phylogenetic and developmental limits of plasticity [59] in an evolutionary context.

For developmental plasticity to occur, organisms must do three things. First, they must detect and integrate environmental cues (such as day-length or temperature) using sensors, such as specific receptor-neuron pairings (sensors; Figure 3). Second, this environmental information must be transmitted to the tissue(s) that should alter their developmental trajectory to align with the new environment. This transmission is done via modulators that often involve neuroendocrine signalling (modulators; Figure 3). Finally, gene expression or gene function (including alternative splicing or post-translational modification of protein) in the affected tissues must be altered to result in the alternative developmental phenotypes (effectors; Figure 3; reviewed in [30,31]).

For many, even well-studied, examples of developmental plasticity, we lack a complete understanding of how developmental plasticity works at a whole organism level. A detailed knowledge of the developmental biology that underpins the different plastic phenotypes is key to generating this integrated understanding. This requires answering a number of questions. When during development are the insects sensitive to the environmental cue? What tissues are affected by the development of the alternative phenotypes? What is the molecular and cellular basis of how these tissues normally develop? This information is only known for some of the examples shown in Figure 2 and is lacking for most other species that exhibit developmental plasticity. This fundamental knowledge is required before targeted experiments can be designed to generate an integrated understanding of how sensors, modulators, and effectors work together to alter developmental pathways resulting in plastic phenotypes.

### Sensors

Developmental plasticity can be triggered by a diverse range of environmental cues (Figure 3). Here, we consider environmental cues as belonging to one of two categories; either ‘generic’ or ‘specialised’. Generic cues, such as temperature, day length, and nutrition can be detected by all insects, whereas specialised cues may be species- or family-specific cues (e.g. pheromones). These two categories of cues raise different questions regarding the evolution and mechanisms of developmental plasticity. For example, for generic cues, why are not all insects that are able to detect the cue also able to exhibit developmental plasticity. Likewise, how does the same cue elicit distinct phenotypes in different species? For specialised cues, these require knowledge of how novel sensor/cue systems evolve and whether plasticity in response to specialised cues use the same modulator and effector systems as those used in response to generic cues. In general, much more is known about how generic environmental cues are detected than about specialised cues. But even then, our mechanistic understanding is limited to a few species. Indeed, much of the existing knowledge comes from studies on *Drosophila melanogaster*. Temperature is sensed by heat and cold receptive neurons in the antennae of *D. melanogaster* adults and via the dorsal and terminal organ ganglia in larvae [32]. It has been suggested that the warm receptors evolved specifically in Diptera, while cold receptors are well-conserved and ancient [33], however there may be some species that have independently evolved heat sensitive neurons (e.g. [34]). Cold receptors are excited by cold and inhibited by heat and the tissues where these cold receptors are expressed vary [35]. For example, adult butterflies express temperature sensitive receptors on their wings and antennae, while adults of the true bug *Rhodnius prolixus*, express temperature sensitive proteins in the rostrum, tarsi, tibial pads, and genitalia [35,36]. Crucially, though, very little is known about how temperature is sensed during embryonic, larval, and pupal stages in species other than *D. melanogaster*. Understanding these temperature sensors’ developmental and tissue-specific expression may allow determination of which species, developmental stages, and tissues are susceptible to temperature. Day length or photoperiod is another important generic environmental cue and is detected by photoreceptors that are present in the compound eyes and ocelli of most insects [37]. Photoperiod is integrated through entrainment of the circadian clock [37], but how photoperiodic information is perceived, stored and acted on during insect development is largely unknown [38,39]. Other environmental cues may also be conveyed optically. In the common bluebottle butterfly (*Graphium sarpedon*), optical signals allow detection of the colour of the substrate, and the larvae adjust pupal colouration accordingly to ensure camouflage [40]. All insects are sensitive to both the quality and quantity of nutrition and deprivation during development results in smaller body size [16]. In insects, nutrient absorption occurs mainly in the mid-gut. In *D. melanogaster*, enteroendocrine cells in the mid-gut act as chemical sensors, causing the secretion of regulatory peptides, and transmitting signals to the brain and other tissues via neurons [41]. Given that these generic environmental cues are received regularly, perhaps even constantly, by insects, this raises the question of how the inputs from these cues are buffered to prevent inappropriate plastic responses, particularly in response to fast-changing or fluctuating environments. There may be multiple mechanisms to differentiate signal from noise and ensure the fidelity of the environmental signal, but the molecular bases of these remains largely unknown [30,42].

Some environmental cues are specialised, and knowledge of developmental plasticity in response to these cues is restricted to a small number of species. For example, pheromones of eusocial insects usually induce species-specific developmental plasticity. One example is the soldier inhibiting pheromone in *Pheidole* ants, which represses the development of more soldiers [43,44]. The exact nature of this pheromone is undetermined [45], but it is likely detected through chemosensory systems like olfactory receptors. The olfactory receptor family is highly divergent amongst insects and evolves rapidly. For many systems, including the soldier inhibiting pheromone in *Pheidole*, more work is required to link pheromones with their cognate receptors [46].

Some examples of developmental plasticity combine specialised species-specific cues with generic cues. For example, *D. melanogaster* larvae are sensitive to the density of conspecific larvae, which has various developmental impacts such as altering adult brain morphology and learning ability [47,48], and testes size [49]. Larvae are attracted to each other through visual cues [50] and pheromones [51]. Combining multiple cues [52] and responding to both species-specific and generic cues adds to the complexity of environmental information gathering and may enhance cue fidelity reducing the chances of mismatch. In cases where developmental plasticity is invoked by combinations of cues [52] more work is needed to determine; how each cue is sensed, the relative importance of each cue in establishing developmental plasticity, and how the information from different cues is integrated and passed to the modulator systems.

There is debate about the exact nature of the inducing environmental cue, even in some well-studied examples of developmental plasticity. Honeybee queen caste development is triggered when royal jelly secreted by workers is fed to developing larvae (Figure 2A). However, the bioactive component(s) of royal jelly remain unknown [53,54], and how royal jelly triggers the developmental trajectories that give rise to the queen and worker phenotypes is yet to be determined [55,56]. Recent studies indicate that the abundance of food provided may drive caste development [57]. However, it remains unclear whether there is a single dominant cue within royal jelly or if multiple cues are acting in concert, perhaps to establish different aspects of queen physiology. Knowing what these cues are will allow the design of targeted experiments to determine the sensor system(s) required to mediate this remarkable example of developmental plasticity.

Establishing an explicit link between an environmental cue and sensor system is non-trivial, but it is a necessary step to design experiments that make reliable predictions and deepen our understanding of developmental plasticity. Understanding the exact links between cues and sensors will allow us to determine if there are any general rules regarding the use of single cues versus multiple cues in developmental plasticity. For example, if abiotic environmental parameters are more likely to use just one detection route and whether this is more likely to involve generic cue-sensor systems. Whereas, biotic cues such as the presence of conspecifics, competitors or predators, may require multiple sensory inputs [58] and involve specialised sensors. These general rules would inform our understanding of how taxonomically constrained plasticity is taxonomic and the developmental limits of plasticity [59]. Defining the cue-sensor systems responsible for developmental plasticity will also allow us to evaluate how the complexity of sensor-cue systems is balanced with the costs of developing and maintaining such systems, illuminating some of the fundamental principles underlying the evolution of plasticity.

### Modulators

Modulators act to link the environmental cue-sensor systems with the tissues that will be affected by developmental plasticity. In insects, the principal modulator is the neuroendocrine signalling system, and this encompasses juvenile hormone (JH), ecdysone, insulin, and biogenic amines (reviewed in [60]). These core components of the neuroendocrine signalling network are involved in many different examples of developmental plasticity. We also know that the function and regulation of these molecules differs between insect species (reviewed in [61]).

Ecdysone signalling during development is implicated in temperature-sensitive wing patterning in butterflies [62] and crowding-induced wing development in aphids [63]. Similarly, JH levels are associated with the development of different castes of eusocial insects, wing and reproductive plasticity in aphids, and plasticity in the size and shape of beetle horns (reviewed in [64]). These modulators do not act in isolation, and there is evidence from several insect species indicating cross-talk between these modulators in different contexts [65]. Aphid wing plasticity is one example of multiple modulators working together to affect developmental plasticity. In this example, nutrition, presumably via insulin signalling, causes a difference in JH sensitivity triggering wing muscle degeneration [66].

This seemingly limited range of available modulators raises the question of how diverse environmental cues can all signal through these components yet give rise to different but specific phenotypes (Figure 3). How this common set of modulators generates the specificity in species and tissue responses is one of the largest outstanding questions in developmental plasticity and is crucial to the understanding of how development is coordinated.

The specificity in responses to environmental cues could be explained by the existence of sensitive and resilient periods in development that differ between developmental stages, sexes, genotypes or species (Figure 1). These sensitive periods may represent critical periods for cell differentiation, tissue growth or tissue patterning. Therefore, only an environmental cue received during this discrete critical period may result in developmental plasticity [67,68].

We hypothesise that the number and length of these critical periods may differ between holometabolous and hemimetabolous insects (Figure 1). In holometabolous insects, patterning during embryonic development establishes the tissues and organs required for larvae, but adult structures are formed during larval development. This means that both patterning and growth processes are potentially sensitive to environmental variation throughout development (Figure 1). Conversely, in hemimetabolous insects, growth and patterning are largely uncoupled; the patterning and formation of adult tissues occurs in the embryo, with growth occurring during the post-embryonic stages independent of further widespread patterning (Figure 1). Generally, it is thought that developmental processes related to growth are more plastic [16,69–71] than those involved in cell fate specification, cell identity, and patterning, which are more robust to environmental perturbation [16,69,70,72]. The robustness in cell fate specification, identity and patterning may be required for generating functional organ structures and viable adults [16,69,70,72]. Growth and patterning often occur simultaneously, and recent studies have begun to address how robustness in pattern is maintained while plasticity in growth occurs [73].

That insects have critical periods for plasticity during development may provide a possible explanation for tissue-specific plasticity. Environmental cues that are received when tissues are being formed during development may result in specific plastic changes to that tissue. However, critical periods alone may not fully explain the tissue-specific responses seen in developmental plasticity. Given the species-specific differences in expression of hormone receptors during development and between tissues [62], it may be that regulation of the hormone receptors also plays a role in mediating tissue-specific responses to environmental cues. There is also extensive cross-talk and feedback within the neuroendocrine system [74,75], raising the possibility that specific combinations of modulators may be critical to mediate tissue specific responses to environmental cues. However, further research is required to determine if or how specific tissues respond to different combinations of modulators and the gene regulatory networks that underpin tissue specific responses to these hormones (effectors).

To address how the neuroendocrine system incorporates diverse environmental information from sensor-cue systems and gives rise to a range of different phenotypes, we firstly need a more fine-grained understanding of the dynamics of changes in neuroendocrine signalling following exposure to a range of environmental cues. Secondly, we need to understand how extensive the cross-talk between the components of the neuroendocrine signalling system is [65] and whether the cross-talk differs between species and/or developmental stages of the same species. This requires sensitive, inexpensive and relatively high-throughput assays to detect hormones and biogenic amines. A range of techniques, including liquid chromatography–mass-spectrometry (LC–MS) [76], gas chromatography–mass spectrometry (GC–MS) and high-performance liquid chromatography (HPLC) are often used to measure JH and ecdysone levels in circulating hemolymph [77]. HPLC is also used to detect biogenic amines [78]. These methods are, however, relatively expensive and require specialised equipment. A commonly used alternative to detect hormone signalling is using RT-qPCR for transcriptional targets of these signalling systems as proxies for the activity of these pathways (Table 1) e.g. *Krüppel homolog1* (*Kr-h1*) is responsive to JH levels [79]. Although these transcriptional proxies of hormone signalling have been used across a wide range of insects, direct links between hormone signalling and transcription of these genes have only been shown for a small number of species, often in the context of metamorphosis rather than developmental plasticity (Table 1). Therefore, a combination of techniques is recommended to characterise the modulators responding to an environmental cue. Characterisation of modulators and how they interpret information from various sensor systems to specifically interface with effectors will be facilitated by technological advancements to increase the sensitivity and decrease the cost of detection in a wide range of species.

**Table 1:** Commonly used transcriptional proxies for modulator (hormone) activity in insects

Symbol	Gene	Description	Signalling pathway	Example references
*Kr-h1*	*Krüppel-homolog 1*	Zinc-finger transcription factor	Primary response to JH	[79,114]
*Br-C (br)*	*Broad-complex (broad)*	Zinc-finger transcription factor	Primary response to 20E	[114]
*E74 (Eip74EF)*	*Ecdysone-induced protein 74EF*	ETS domain transcription factor	Primary response to 20E	[115]
*E75 (Eip75B)*	*Ecdysone-induced protein 75B*	Nuclear receptor that interacts with Hormone receptor 3 (Hr3)	Primary response to 20E	[116]
*E93 (Eip93F)*	*Ecdysone-induced protein 93F*	Helix–turn–helix transcription factor	Primary response to 20E (may be repressed by JH)	[117]
*4-EBP (Thor)*	*Translation initiation factor 4E binding protein*	Contributes to translation regulation	Insulin signalling (target of the FOXO transcription factor)	[118]
*bmm*	*Brummer*	Triglyceride lipase involved in glycerolipid metabolism	Insulin signalling (target of the FOXO transcription factor)	[119,120]
*ILP6*	*Insulin-like peptide 6*	Insulin receptor binding activity	Insulin signalling (target of the FOXO transcription factor)	[121]
*InR*	*Insulin-like receptor*	Receptor protein-tyrosine kinase	Insulin signalling (target of the FOXO transcription factor)	[122]

### Effectors

The ultimate effectors of developmental plasticity are genes and gene regulatory networks that mediate differences in cell growth, cell death, or patterning and give rise to markedly different plastic phenotypes. These effector modules are manifest as tissue-specific gene expression patterns at key points in development as the alternative phenotypes are being established. There are a large number of studies that have focussed on examining tissue-specific changes in gene expression during developmental plasticity in a wide range of insect species (e.g. [55,80], reviewed in [31]). These studies have identified various genes, including transcription factors, that may be key to establishing plastic phenotypes (e.g. [55,80,81–85]). Given the diversity of phenotypes induced by developmental plasticity and the range of tissues and cell types affected (Figure 2), it seems logical to suggest that there would be little overlap in the effectors (the genes and gene regulatory networks) that underpin them. However, a comprehensive meta-analysis of published studies would be required to assess this empirically. There is relatively little overlap in studies that assessed overlap in differentially expressed genes between similar examples of developmental plasticity. For instance, despite caste in bumble bees and honeybees being regulated by a common modulator (JH) [64], there is relatively little overlap in the expression of downstream effectors between these two species [86]. Similarly, density-dependent locust phase polyphenisms are regulated by almost entirely different sets of effectors, even in closely related species (6 million years diverged) [87]. The observed diversity of effectors does, however, reinforce the question of how such a limited set of modulators can act in such a tissue specific way. To address this question, we need to link the modulators directly with the key effectors in a tissue, the genes that are responding to the modulators and facilitating changes in tissue development.

Identifying key effectors of developmental plasticity in large lists of differentially expressed genes can be challenging. Many studies have relied on gene ontology, pathway or network analysis to assist, (e.g. [13,55,80,88,89]). These approaches have been successful (e.g. [13,55,80,88,89]) but rely on one-to-one homology and assume conservation of function between *D. melanogaster* and the species of interest. These analyses, therefore, omit rapidly-evolving genes and recent gene duplications [90], which may not have an identifiable ortholog in *D. melanogaster*.

Identifying key effectors of developmental plasticity in species of interest has, however, been transformed by the development of Assay for Transposase-Accessible Chromatin followed by high-throughput sequencing (ATAC-seq), which can be used to determine accessible *cis*-regulatory regions [91], transcription factor binding sites [92], and actively bound transcription factors within open regions of chromatin. Using techniques such as ATAC-seq, it is now possible to understand how gene regulation differs between phenotypes in developmental plasticity and use this as a tool to identify key effectors, such as transcription factors, of the different phenotypes.

Another major advance is single-cell RNA-seq (scRNA-seq) [93] which allows profiling of the most highly expressed transcripts in each cell type. scRNA-seq will be particularly useful for studies of developmental plasticity as it will allow us to determine if developmental plasticity is resulting in the expansion of particular cell populations within an embryo, larvae, pupae, or tissue. It also allows us to determine whether developmental plasticity is altering cell-fate specification or patterning, and to identify which cells within a developing organism are responding to the environmental stimulus via modulators. Thus, adding a layer of spatial understanding into these gene expression studies that is not possible with bulk RNA-seq.

Although gene expression is likely to be the biggest driver of the development of alternative phenotypes [94], epigenetic modifiers, such as DNA methylation, have been implicated in phenotypic plasticity. In insects, DNA methylation doesn't have a consistent role in developmental plasticity and is not associated with altered gene expression (reviewed in [95]). Other epigenetic modifications, such as histone modifications, may have a role in stabilising differences in gene expression [96], and their role in developmental plasticity is an active area of research, e.g. [97].

Once candidate key effectors have been identified, manipulative studies are required to differentiate those genes associated with developmental plasticity from key effectors that are orchestrating the phenotype. Manipulative studies are now possible in a wide range of non-model insect species. They may use RNA interference to reduce gene expression, chemical inhibitors to inhibit particular pathways or CRISPR/Cas9 genome editing. Such manipulative studies, combined with measuring the trait of interest under at least two environmental conditions, are essential to demonstrate the role of a particular gene or pathway in developmental plasticity. These approaches require a thorough understanding of the expected timescales and extent of change to appropriately interpret the phenotype(s) generated by these manipulative studies.

## An integrated evo-devo approach to developmental plasticity

Developmental plasticity could evolve through modification of sensor, modulator, or effector; e.g. a novel sensor could allow a species to become sensitive to a new environmental cue, modulators could respond differently to the cue, or effector systems could be co-opted to respond to these modulators. However, as yet, it is unclear whether any of these components are more evolutionarily labile. To determine if we can make generalisations about how plasticity evolves, we need to understand how it works and how it is encoded in the genome in a range of phylogenetically diverse species. Unfortunately, there are currently very few examples of plasticity where we can link sensors, modulators and effectors to a developmental outcome. However, one elegant example is seen in the butterfly *B. anynana*.

Many *Bicyclus* genus and Nymphalid butterflies exhibit prominent eye spots on their wings [98], and in some species, the size of the eyespots is developmentally plastic ([99,100], reviewed in [101]). In *B. anynana*, large exposed ventral eyespots are observed in the wet season and smaller eyespots are observed in the dry season [100]. The plasticity in eyespot size appears to be adaptive, with large exposed ventral eyespots directing predator attacks to the wing margins in the wet season [17,102,103] and the smaller eyespots helping camouflage the butterflies and protect against predation in the dry season [17]. This developmental plasticity in eyespot size is tightly linked to temperature, a temperature of over 25°C is associated with large eyespots, while below 20°C leads to smaller eyespots [100,104]. At these temperatures there are differences in the amount of 20-hydroxyecdysone (20E) that are detected during the wandering stages of larval development [105], and a shift in the dynamics of 20E titre during pupal development [106,107]. However, how temperature is sensed and the molecular details of how temperature links to 20E titre is still to be determined in this species [101]. Intriguingly, the levels of 20E also respond to temperature in a range of Nymphalid species including those without plasticity in eyespot size [62]. 20E signals through the ecdysone receptor and only species with complex eye spots express the ecdysone receptor in the developing wing, but they do so whether or not there is plasticity in eyespot size [62]. This suggests that developmental plasticity in *B. anynana* has evolved via changes in the effector system rather than sensor or modulator systems. Indeed, the eyespots of *B. anynana* are differentially sensitive to 20E compared with other, relatively closely related, species [62]. Furthermore, in *B. anynana* this differential sensitivity to 20E is associated with cell division and larger eyespots [108]. Using this approach of comparing wing eyespot development in closely related species is incredibly powerful in terms of delineating the mechanisms of developmental plasticity. This comparative work is also important for understanding the evolution of developmental plasticity as although eyespot size plasticity associated with seasons is widely conserved across Nymphalinae butterflies [109], it has become clear from this work that this plasticity has evolved independently multiple times and is regulated by different mechanisms [62]. This example demonstrates how powerful the evo-devo framework is for identifying the mechanisms underlying developmental plasticity.

By developing an integrated understanding of how developmental plasticity works in individual species, how developmental plasticity and polyphenisms have evolved can be addressed. Much of what is currently known about the mechanisms of developmental plasticity in insects comes from studies of polyphenisms. Yet it is currently unknown whether the mechanisms (sensors, modulators, and effectors) that govern polyphenisms are the same as those that regulate examples of continuous developmental plasticity (Figure 2F). Additionally, it remains unclear how these developmental processes are canalised in polyphenisms to robustly produce only two discrete phenotypes without intermediates.

If we can understand how plasticity works and how it evolved in a phylogenetically diverse range of species that span the 475 my of insect evolution [110] and encompassing a range of different phenotypes and different environmental cues, we will ultimately be able to determine if there are any commonalities in independently evolved instances of developmental plasticity. This will allow determination of whether general rules about developmental plasticity exist, allowing us to predict which species have plasticity in particular traits, or in response to certain cues, what the developmental limits of that plasticity might be and whether plasticity may provide resilience to some species in the face of global change [52,111–113].

## Perspectives

- Developmental plasticity, the ability to generate two or more phenotypes from the same genome in response to an environmental cue during development, is taxonomically widespread throughout plants and animals. Understanding how developmental plasticity works and how it evolves will not only transform our fundamental understanding of development and evolution, but also have practical implications for assessing whether plasticity can act as a rapid response mechanism to rapid or fluctuating environmental changes and facilitate species resilience.
- For developmental plasticity to function, insects must detect the environmental cue using sensors. This information is then passed to the tissue that will be altered by modulators, primarily the neuroendocrine system. Modulators then activate effectors, such as a gene expression programme, to trigger different developmental trajectories. An integrated understanding of how developmental plasticity works for most organisms is lacking. In particular, we don't yet understand how different environmental cues can all be interpreted through the same modulatory system to generate different phenotypes. Understanding this will identify key rules of life and tell us how developmental plasticity evolved.
- Future work should focus on (1) more detailed and integrated understanding of developmental plasticity for established model species, (2) determining direct casual links between genes and developmental phenotypes, and (3) incorporating a phylogenetically diverse range of species to broaden our understanding of how developmental plasticity works and how it evolved. For established model species with sequenced genomes, incorporating new technical approaches can increase the granularity of our mechanistic understanding. Two techniques currently standout for this; ATAC-seq and single cell RNA-seq. ATAC-seq can link how effector modules are regulated to the differential gene expression that usually underpins phenotypic variation. While single-cell/tissue specific RNA-seq allows for greatly increased specificity to allow investigators to more clearly link variation of gene expression with phenotypic variation within tissue that are known to influence the phenotype of interest. Both of these approaches require high quality genomic resources. Testing the function of key sensors, modulators, and effectors can be achieved with RNAi or CRISPR/Cas9 genome editing. RNAi is well-established in a number of insect species and can be used without the need to maintain mutant strains when a gene/species is responsive to this manipulation. CRISPR/Cas9 genome editing provides a way to generate null mutations for any species, but has very low penetrance and requires mutant strains to be kept and bred. Both techniques require knowledge of the target sequence, extensive validation, and experimental controls to ensure correct targeting and penetrance. Experimental design should ensure adequate statistically powered design, factorial designs are preferred compared with two single factor designs, and ensure biological replication is done at an appropriate level for the question asked (i.e. ensuring replicates are genetically diverse enough for the question; using only one genetic strain can limit the scope of the conclusions). Using diverse species is necessary to achieve a broad understanding of any phenomenon and what elements underpinning a phenotype are general and which are species specific. This will be facilitated by employing an evo-devo framework that compares species that exhibit plasticity and closely related species that do not.

## Open Access

Open access for this article was enabled by the participation of University of Leeds in an all-inclusive *Read & Publish* agreement with Portland Press and the Biochemical Society under a transformative agreement with JISC.

## Abbreviations

ATAC-seq: Assay for Transposase-Accessible Chromatin followed by high-throughput sequencing
HPLC: high-performance liquid chromatography
JH: juvenile hormone
